# Antimicrobial synergy of cationic grafted poly(*para*-phenylene ethynylene) and poly(*para*-phenylene vinylene) compounds with UV or metal ions against *Enterococcus faecium*

**DOI:** 10.1039/c8ra02673d

**Published:** 2018-06-27

**Authors:** Jordan McBrearty, David Barker, Mona Damavandi, Joels Wilson-Nieuwenhuis, Lisa I. Pilkington, Nina Dempsey-Hibbert, Anthony J. Slate, Kathryn A. Whitehead

**Affiliations:** Microbiology at Interfaces Group, Faculty of Science and Engineering, Manchester Metropolitan University Manchester M1 5GD UK K.A.Whitehead@mmu.ac.uk +44(0) 161 247 1157; School of Chemical Sciences, University of Auckland Auckland 1010 New Zealand

## Abstract

The rise in multidrug resistant bacteria is an area of growing concern and it is essential to identify new biocidal agents. Cationic grafted compounds were investigated for their antimicrobial properties using minimum inhibitory concentration (MIC) and minimum bactericidal concentration (MBC) tests. Synergy testing was carried out using the compounds in the presence of ultraviolet (UV). Fractional inhibitory concentration (FIC) and fractional bactericidal concentration (FBC) tests were carried out using the cationic molecules in conjunction with metal ion solutions of gold, silver, palladium, platinum, rhodium, titanium, tin, vanadium and molybdenum. Individually, the cationic compounds containing quaternary amines, polyphenylene vinylene (PPV) with long polyacrylate grafts (PPV-*g*-PMETAC (HMw)), polyphenylene ethylene (PPE) with long polyacrylate grafts (PPE-*g*-PMETAC (HMw)), polyphenylene vinylene (PPV) with short polyacrylate grafts (PPV-*g*-PMETAC (LMw)) and polyphenylene ethylene (PPE) with short polyacrylate grafts (PPE-*g*-PMETAC (LMw)) were effective against *Enterococcus faecium*. The most successful compound under UV was PPV-*g*-PMETAC (HMw). Following the FICs, palladium and rhodium ion solutions caused a synergistic reaction with all four tested compounds. The presence of conjugated bonds in the cationic molecules increased its antimicrobial activity. These results suggest that the chemical backbone of the compounds, alongside the chain lengths and chain attachment affect the antimicrobial efficacy of a compound. These factors should be taken into consideration when formulating new biocidal combinations.

## Abstract 2

The rise in multidrug resistant bacteria is an area of growing concern and it is essential to identify new biocidal agents.

## Graphical abstract

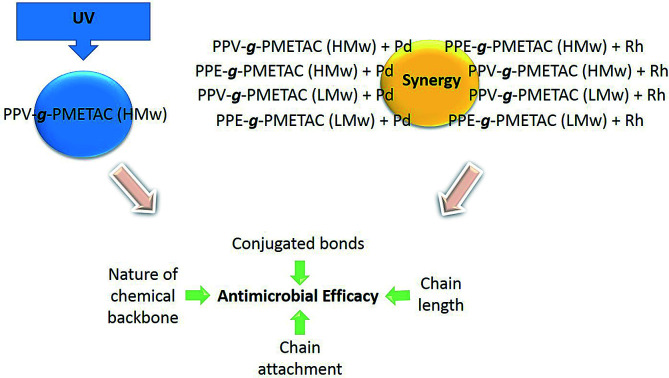

## 1. Introduction

Antimicrobial resistance (AMR) is a serious worldwide issue, with our ability to treat many infections becoming increasingly difficult. Cationic antimicrobials have been widely deployed in antisepsis for well over half a century without any apparent reduction in their effectiveness.^1^ Cationic compounds have an overall positive charge, which influences the interactions between the molecule and the bacterial cells.^2^ Conjugated polyelectrolytes (CPEs) have attracted much attention in recent years as a new class of materials.^3–5^ Within the diverse categories of synthesised CPEs, modified-charge CPEs have been shown to demonstrate antibacterial efficiency, which has been attributed to their structure.^6–9^

The antimicrobial ability of compounds can be altered or improved by the manipulation of specific variables or by using them in combination with other treatments to yield synergistic effects. Photo-stimulation can be used to activate some classes of compounds, resulting in chemical reactions that make the molecules more biologically active. The utilization of light to activate or enhance the biocidal activity of compounds generally results in a process that causes a phototoxic reaction, resulting in the release of a reactive oxygen species, leading to cell damage or death.^10,11^ Metals are known to have antimicrobial properties and silver is arguably the best known antimicrobial metal, although its activity varies in different forms.^12,13^ Gold complexes have historically been investigated as antimicrobial compounds showing excellent activity against wound and other skin related infections.^14^ Palladium, platinum and rhodium are all platinum group metals which share similar physical and chemical properties resulting in similar antimicrobial potentials.^15,16^ Titanium, tin, gallium and molybdenum have also all been shown to have antimicrobial potential in various forms.^17–19^ Vanadium-based compounds have previously displayed antimicrobial properties in vanadium peroxidase reactions against *Streptococcus mutans* planktonic and biofilm morphologies.^20^

*Enterococcus faecium* is a commensal bacteria that is usually a part of the gut micro flora. It is also an opportunistic pathogen, often reported as the causative agent of hospital acquired infections worldwide.^21^*E. faecium* is one of the top causes of nosocomial infections and the use of novel biocides to enable its effective eradication from hospital surfaces and reduce its transmission would be advantageous. This is especially important due to the AMR displayed by this particular bacterial species to a wide range of compounds. The aim of this proof-of-concept study was to test the antimicrobial efficacy of modified polymeric polyphenylene ethynylene (PPE) and polyphenylene vinylene (PPV) compounds with high and low molecular weight side chains against *E. faecium*. The activity of these compounds in the presence of UV or in conjunction with metal ion solutions was also tested to look for synergistic antimicrobial interactions. It was envisaged that such combinations may be advantageous in reducing healthcare related infections.

## 2. Material and methods

**Scheme 1:**
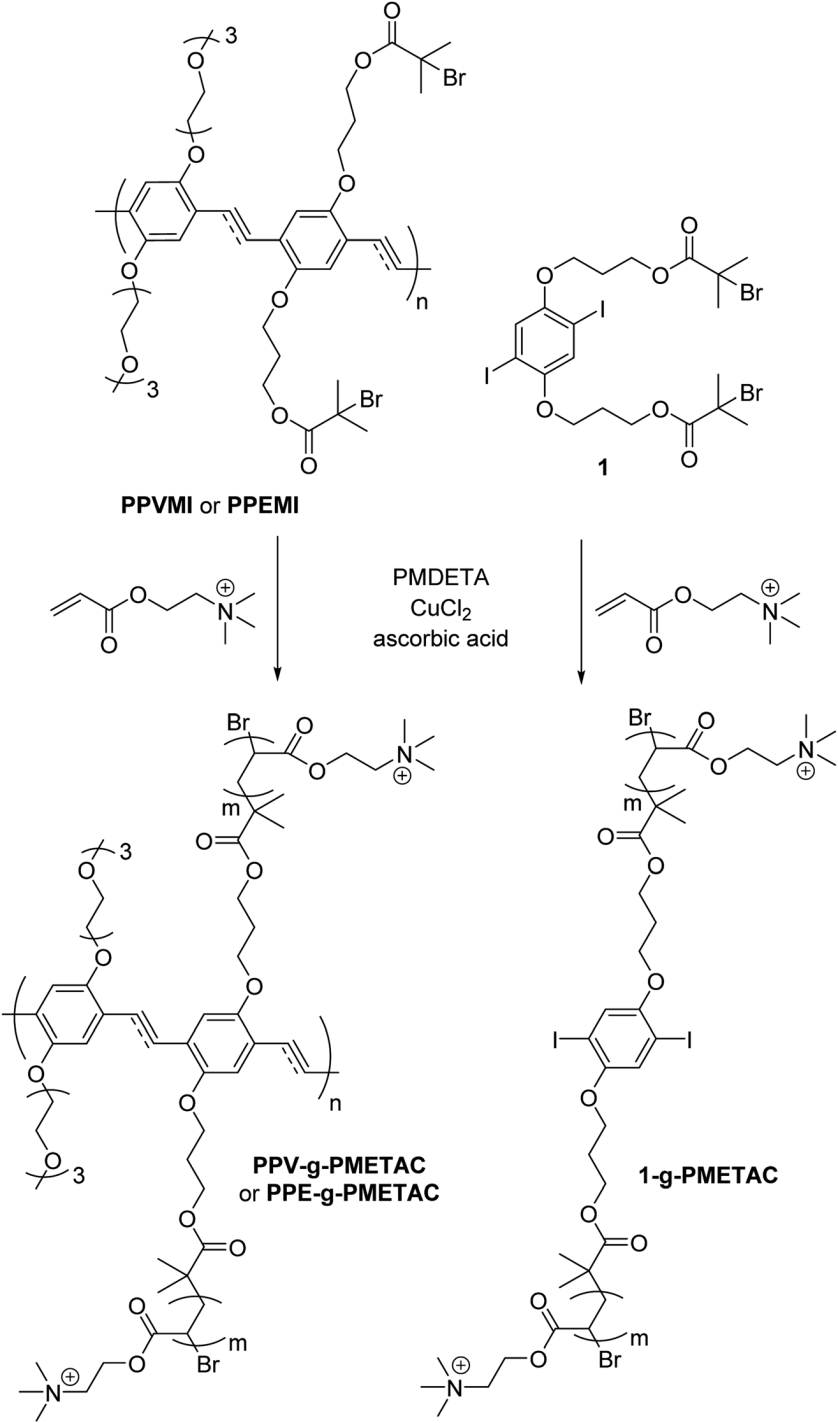
Synthesis of PPV-*g*-PMETAC, PPE-*g*-PMETAC and 1-*g*-PMETAC.

The compounds used in this study were produced using Activator ReGenerated by Electron Transfer Atom Transfer Radical Polymerisation (ARGET ATRP) (Scheme 1).^22,23^

### 2.1 Synthetic methods to prepare anti-bacterial polymers

((2,5-Diiodo-1,4-phenylene)bis(oxy))bis(propane-3,1-diyl) bis(2-bromo-2-methylpropanoate) 1 was prepared as follows. 4-Dimethylaminopyridine (DMAP) (28 mg, 0.23 mmol) followed by α-bromoisobutyryl bromide (140 μL, 1.13 mmol) was added dropwise to a solution of 3,3′-((2,5-diiodo-1,4-phenylene)bis(oxy))bis(propan-1-ol) (200 mg, 0.42 mmol) in dry dimethylformamide (DMF) (4 mL) and triethylamine (200 μL) and was stirred at 0 °C, then warmed to room temperature over 48 h.^22^ The mixture was filtered, washed with water (15 mL), NH_4_Cl (15 mL), NaHCO_3_ (15 mL) and brine (15 mL) then dried over sodium sulphate. The solvent was removed under vacuum and the mixture was purified using flash chromatography (*n*-hexanes-EtOAc, 9 : 1) to give 1 (0.134 g, 75%) as a white solid. The spectroscopic data matched that previously reported in literature.^22^

### 2.2 Preparation of copolymer 1-*g*-PMETAC

The solution mixture of dibromide 1 (15 mg, 0.02 mmol) in DMSO (5 mL) was added to a solution of [2-(methacryloyloxy)ethyl]trimethylammonium chloride (METAC) (1.88 mL, 10 mmol) in DMSO (10 mL) and water (0.6 mL) and left at room temperature until a clear solution was obtained. The ligand–catalyst complex was prepared by adding *N*,*N*,*N*′,*N*′′,*N*′′-pentamethyldiethylenetriamine (PMDETA) (7.5 mg, 0.041 mmol) to Cu(ii)Cl (2 mg, 0.0148 mmol) in anisole (1 mL) at 67 °C. The ligand–catalyst complex was added to the reaction mixture and heated to 60 °C. A mixture in anisole (1 mL) of ascorbic acid (980 mg, 5.56 mmol) and water (0.30 mL) was added slowly to the reaction and left under an atmosphere of nitrogen at 60 °C for 24 h. The reaction was quenched with exposure to air and by cooling the reaction flask in liquid nitrogen. The precipitated grafted polymer was filtered and dissolved in water (5 mL). The grafted polymer was re-precipitated into acetone (100 mL) and centrifuged to collect the product as a white solid (78 mg, 42%). *δ*_H_ (300 MHz; D_2_O): 4.35–4.52 (2H, m, CH_2_); 3.70–3.77 (2H, m, CH_2_); 3.22 (9H, s, CH_3_); 1.95–2.02 (1H, m, CH); 1.31–1.55 (2H, m, CH_2_). GPC: *M*_w_: 6.41 × 10^3^, *M*_n_: 4.98 × 10^3^, *M*_w_/*M*_n_: 1.29.

### 2.3 Preparation of copolymers PPV-*g*-PMETAC (HMw) and PPV-*g*-PMETAC (LMw) from PPVMI

**Fig. 1:**
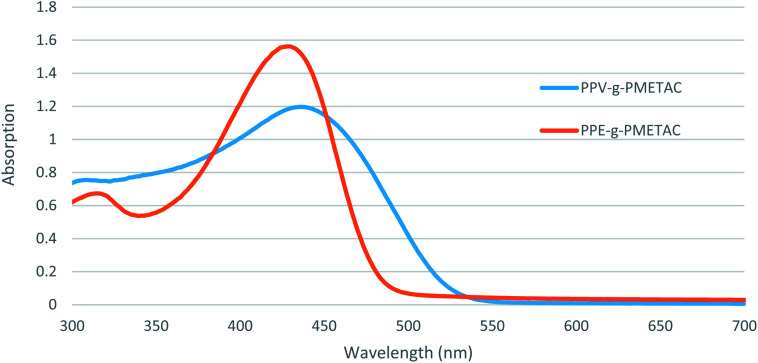
UV-Vis absorption spectra of PPV-*g*-PMETAC (LMw, light grey) and PPE-*g*-PMETAC (dark grey).

A solution of PPVMI (270 mg, 0.01 mmol) in DMSO (5 mL) was added to a stirring solution of METAC (1.88 mL, 10 mmol) in DMSO (5 mL) and water (600 μL) to achieve a colourless solution.^23^ Separately the ligand–catalyst complex was prepared by adding PMDETA (7.5 mg, 0.041 mmol) into a mixture of Cu(ii)Cl (2 mg, 0.0148 mmol) mixture in anisole (1 mL) at 67 °C for 3 h. This complex was added to the reaction mixture at 60 °C. A solution of ascorbic acid (980 mg, 5.56 mmol) in anisole (1 mL) and water (300 μL) was added slowly to the reaction and left for 24 h under an atmosphere of nitrogen at 60 °C. The reaction was quenched by cooling the mixture with liquid nitrogen and then exposing the mixture to air. The orange precipitate was collected and dissolved in water, then was re-precipitated using acetone (100 mL) and collected using centrifugation to produce a bright orange solid PPV-*g*-PMETAC (HMw) (850 mg, 55%). *δ*_H_ (400 MHz; D_2_O): 1.01–1.10 (2H, m, CH_2_), 1.95–2.01 (1H, m, CH), 3.25 (9H, br s, CH_3_), 3.75–3.82 (2H, m, CH_2_), 4.45–4.50 (2H, m, CH_2_). GPC: *M*_w_: 77.50 × 10^3^, *M*_n_: 46.19 × 10^3^, *M*_w_/*M*_n_: 1.68. *λ*_max abs_ = 435 nm. *λ*_max em_ = 535 nm. The same procedure was used to achieve the low molecular weight polymer, except different amounts of the chemicals were used; METAC (950 μL, 5 mmol) in DMSO (5 mL) and water (300 μL) with ascorbic acid (600 mg, 3.40 mmol) in anisole (1 mL) and water (150 μL) to give a bright orange solid PPV-*g*-PMETAC (LMw) (180 mg, 40%). *δ*_H_ (400 MHz; D_2_O): 1.01–1.10 (2H, m, CH_2_), 1.92–2.03 (1H, m, CH), 3.28 (9H, br s, CH_3_), 3.77–3.85 (2H, m, CH_2_), 4.43–4.48 (2H, m, CH_2_). GPC: *M*_w_: 22.87 × 10^3^, *M*_n_: 16.72 × 10^3^, *M*_w_/*M*_n_: 1.37. *λ*_max abs_ = 435 nm. *λ*_max em_ = 537 nm (Fig. 1).

### 2.4 Preparation of copolymers PPE-*g*-PMETAC (HMw) and PPE-*g*-PMETAC (LMw) from PPEMI

A solution of PPEMI (220 mg, 0.02 mmol) in DMSO (5 mL) was added to a stirring solution of DMSO (5 mL) and water (600 μL) containing METAC (1.88 mL, 10 mmol) to achieve a colourless solution.^23^ Separately, the ligand–catalyst complex was prepared by adding PMDETA (7.5 mg, 0.041 mmol) into a mixture of Cu(ii)Cl (2 mg, 0.0148 mmol) in anisole (1 mL) for 3 h at 67 °C. This complex was added to the reaction mixture at 60 °C. A solution of ascorbic acid (980 mg, 5.56 mmol) in anisole (1 mL) and water (300 μL) was added slowly to the reaction and left at 60 °C for 24 h under an atmosphere of nitrogen. The reaction was quenched by cooling the mixture with liquid nitrogen and exposing the mixture to air. The orange precipitate was collected and then dissolved in water, then was re-precipitated using acetone (100 mL) and collected using centrifuge to achieve a bright orange solid PPE-*g*-PMETAC (HMw) (870 mg, 63%). *δ*_H_ (400 MHz; D_2_O): 1.02–1.11 (2H, m, CH_2_), 1.97–2.03 (1H, m, CH), 3.26 (9H, br s, CH_3_), 3.77–3.82 (2H, m, CH_2_), 4.47–4.51 (2H, m, CH_2_). GPC: *M*_w_: 77.48 × 10^3^, *M*_n_: 46.03 × 10^3^, *M*_w_/*M*_n_: 1.68. *λ*_max abs_ = 428 nm. *λ*_max em_ = 493 nm. The same procedure was used to achieve the low molecular weight polymer, except using METAC (950 μL, 5 mmol) in DMSO (5 mL) and water (300 μL) and a solution of ascorbic acid (600 mg, 3.40 mmol) in anisole (1 mL) and water (150 μL) to give a bright orange solid PPE-*g*-PMETAC (LMw) (190 mg, 43%). *δ*_H_ (400 MHz; D_2_O): 1.04–1.13 (2H, m, CH_2_), 1.95–2.01 (1H, m, CH), 3.24 (9H, br s, CH_3_), 3.78–3.83 (2H, m, CH_2_), 4.45–4.47 (2H, m, CH_2_). GPC: *M*_w_: 21.97 × 10^3^, *M*_n_: 16.20 × 10^3^, *M*_w_/*M*_n_: 1.36. *λ*_max abs_ = 428 nm. *λ*_max em_ = 497 nm.

For use in the assays, polyphenylene vinylene (PPV) and polyphenylene ethynylene (PPE) polymers were suspended in undiluted water, except for the ungrafted PPV and PPE which were suspended in undiluted DMSO. The composition of the compounds is described (Table 1).

**Table 1:** Descriptions of the compounds used in this study

Class	Number	Structure
Cationic compounds	1-*g*-PMETAC	Non-conjugated polymer (NCP) side chains (quaternary amine acrylic (cationic)) were grafted, not conjugated
	PPV-*g*-PMETAC (HMw)	Polyphenylene vinylene (PPV) with long polyacrylate grafts containing quaternary amines
	PPE-*g*-PMETAC (HMw)	Polyphenylene ethylene (PPE) with long polyacrylate grafts containing quaternary amines
	PPV-*g*-PMETAC (LMw)	Polyphenylene vinylene (PPV) with short polyacrylate grafts containing quaternary amines
	PPE-*g*-PMETAC (LMw)	Polyphenylene ethylene (PPE) with short polyacrylate grafts containing quaternary amines
Neutral compounds	*p*-Phenylene vinylene	PPV: ungrafted polyphenylene vinylene
	*p*-Phenylene ethylene	PPE: ungrafted polyphenylene ethylene

### 2.5 Cultures and media

Stock cultures of *E. faecium* NCTC 7171 were sub-cultured onto columbia blood agar (Oxoid, UK) with defibrillated horse blood (TCS Biosciences Ltd, UK). Bacterial cultures were grown in a 5% CO_2_ incubator for 24 h at 37 °C. Brain heart infusion (BHIA) agar (Oxoid, UK) and brain heart infusion broth (BHIB) (Oxoid, UK) were used for all the microbiological tests.

### 2.6 Bacterial preparation

A single colony of *E. faecium* was inoculated from a BHIA plate into 10 mL of BHIB. The inoculated culture was incubated in a 5% CO_2_ incubator without shaking for 24 h. Cells were harvested by centrifugation (567*g* for 10 min) and then washed with 10 mL sterile distilled water (dH_2_O) and vortexed. The washed cells were re-centrifuged and the pellet was re-suspended in 10 mL of dH_2_O, vortexed and the resultant cell suspension was adjusted to an optical density (OD) at 540 nanometres (nm) (OD_540_) of 1.0 (±0.05) using a spectrophotometer. The cell concentration corresponded to 3.95 × 10^8^ colony-forming units per mL (CFU mL^−1^) at an OD_540_ of 1.0.

### 2.7 Minimal inhibitory concentrations (MIC) and minimal bactericidal concentrations (MBC) assays

A culture of *E. faecium* was prepared and triphenyl tetrazolium chloride (TTC) (Sigma-Aldrich, UK) was added to obtain a working concentration 0.15 w/v. To a 96 well flat-bottomed micro titre plate, 100 μL of antimicrobial sample was added to the appropriate row/column. One-hundred micro litres of the bacterial suspension with the TTC was added to the wells to be tested. Using a multi-channel pipette the first column was mixed, then 100 μL of the sample/bacterial mix was transferred to the next column and mixed until completion until the final column whereby 100 μL was discarded. One-hundred micro litres of the bacterial suspension without the test sample was used as a positive control and 100 μL of sterile double strength broth was used as a negative control. Micro titre plates were incubated in a 5% CO₂ incubator at 37 °C for 24 h. After incubation, the MIC was taken as the lowest concentration that inhibited the visible growth of the bacteria by comparison with the controls (wells turned dark blue/purple indicating growth). The MBC was determined by removing 25 μL of culture from each well that showed no growth. This was pipetted onto agar and incubated in a 5% CO₂ incubator at 37 °C for 24 h. The colonies were counted and calculated to give the CFU mL^−1^. After incubation, the lowest concentration well sample that showed no bacterial growth on the agar plate was determined to be the MBC for that test sample (*n* = 3).

### 2.8 UV MIC and MBC assays

MIC and MBC tests were performed after incubation of the test compounds under UV (UVP Blak-ray, US 365 nm wavelength) at 3 time points, 30 min, 60 min and 90 min (*n* = 3).

### 2.9 Fractional inhibitory (FIC) and fractional bactericidal concentration (FBC) assays

The MICs of the metal ion solution synergies were determined using FIC antimicrobial screening. The metal ion solutions were obtained from Sigma, (UK). The bacterial suspension and test samples for the FIC test were prepared as described in the section bacterial preparation and MIC method, except that the compound and metal ion solutions were added to the wells in a 1 : 1 ratio (*n* = 3). The prepared solutions were homogeneous with no apparent aggregates formed. The same method was carried out for the FBCs except that the bactericidal concentrations were used.

Following incubation at 37 °C for 24 h, the FIC values were calculated as; 1
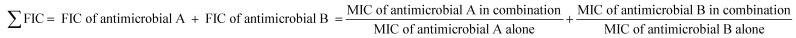


Depending on the FIC values, the antimicrobial interaction was evaluated as synergy = ≤0.5, additive = >0.5 ≤ 1.0, indifference = <1.0 ≤ 4.0 or antagonism = >4.0.

### 2.10 Statistical analysis

Mean values were used to compare the antimicrobial efficacy results. Standard errors were calculated to analyse the distributions of the data from the mean value, and confidence intervals of 95% were calculated to compare the significance of the results.

## 3. Results

### 3.1 MIC of novel compounds with and without UV

When the compounds were tested against *E. faecium*, it was demonstrated that all the grafted poly(*para*-phenylene ethynylene) (PPE) or poly(*para*-phenylene vinylene) (PPV) compounds demonstrated greater antimicrobial activity compared to the ungrafted PPE (1250 μg mL^−1^) or PPV (1250 μg mL^−1^) alone (Table 2). The non-conjugated cationic polymer, 1-*g*-PMETAC, demonstrated the least antimicrobial activity at 2500 μg mL^−1^, suggesting that, against *E. faecium*, the presence of conjugated bonds in the molecule increased its antimicrobial activity. Of the conjugated molecules, PPV-*g*-PMETAC (HMw) demonstrated the best antimicrobial activity (65.1 μg mL^−1^), whilst PPE-*g*-PMETAC (HMw), PPV-*g*-PMETAC (LMw) and PPE-*g*-PMETAC (LMw) demonstrated inhibitory effects at 104.2 μg mL^−1^ (Table 2).

**Table 2:** MIC (μg mL^−1^) of the novel cationic compounds before and after being incubated under UV for 30 min, 60 min and 90 min. MIC indicates the lowest concentrations where inhibition effects were observed

UV duration	1-*g*-PMETAC	PPV-*g*-PMETAC (HMw)	PPE-*g*-PMETAC (HMw)	PPV-*g*-PMETAC (LMw)	PPE-*g*-PMETAC (LMw)	Ungrafted PPV	Ungrafted PPE	DMSO
0 min	2500	65.1	104.2	104.2	104.2	1250	1250	1250
30 min	2500	39.1	78.1	78.1	78.1	1250	1250	1250
60 min	2500	39.1	78.1	156.3	78.1	1250	1250	1250
90 min	5000	78.1	156.3	156.3	78.1	1250	1250	1250

To determine whether any of the compounds were potentially UV activated, MICs were repeated with 30 min, 60 min and 90 min exposure to UV prior to testing (Table 2). After 30 min of UV exposure all the compounds demonstrated an increase in antimicrobial efficacy, with PPV-*g*-PMETAC (HMw) once again being the most active (39.1 μg mL^−1^). The PPE-*g*-PMETAC (HMw), PPV-*g*-PMETAC (LMw) and PPE-*g*-PMETAC (LMw) all demonstrated improved activity compared to the control with MICs at 78.1 μg mL^−1^. After 60 min of UV exposure, the lowered MIC results were the same as those demonstrated following 30 min of UV, with the exception of the PPV-*g*-PMETAC (LMw), which required a higher concentration to inhibit growth (156.3 μg mL^−1^). After 90 min of UV exposure, only the PPE-*g*-PMETAC (LMw) demonstrated an MIC better than the control (78.1 μg mL^−1^). Overall, the cationic molecules with the longest side chains, PPV-*g*-PMETAC (HMw) and PPE-*g*-PMETAC (HMw), demonstrated greater antimicrobial activity than the cationic molecules with the shorter side chains in the presence of UV. Throughout the work, the ungrafted PPE and PPV did not demonstrate any enhancement by UV, whilst the 1-*g*-PMETAC required a greater concentration following 90 min UV irradiation from 2500 μg mL^−1^ to 5000 μg mL^−1^ (Table 2).

### 3.2 MBC of novel compounds (with and without UV radiation)

Similarly to the MIC results, the MBC results demonstrated that PPV-*g*-PMETAC (HMw) had the greatest bactericidal potential (625 μg mL^−1^), followed by PPE-*g*-PMETAC (HMw) (833.3 μg mL^−1^) (Table 3). Both PPV-*g*-PMETAC (LMw) and PPE-*g*-PMETAC (LMw) also demonstrated bactericidal potential (1250 μg mL^−1^), but were less effective. None of the remaining compounds, 1-*g*-PMETAC, the ungrafted PPV and PPE or the DMSO demonstrated any bactericidal properties, even when exposed to UV treatment. In contrast, PPV-*g*-PMETAC (HMw) demonstrated an increase in bactericidal properties (312.5 μg mL^−1^) after 30 and 60 min UV and a further increase after 90 min (156.3 μg mL^−1^) (Table 3). However, PPE-*g*-PMETAC (HMw), PPV-*g*-PMETAC (LMw) and PPE-*g*-PMETAC (LMw) demonstrated a reduction or no change in their bactericidal properties after 30 or 60 min UV exposure. Only after 90 min did PPE-*g*-PMETAC (HMw) and PPE-*g*-PMETAC (LMw) demonstrate an increase in bactericidal properties (625 μg mL^−1^) (Table 3). Taken together, HMW conjugated compounds had the greatest bactericidal potential on their own and in the presence of UV, and UV was shown to increase the antimicrobial efficacy of PPV-*g*-PMETAC (HMw) in the short term.

**Table 3:** MBC (μg mL^−1^) of the novel cationic compounds before and after being incubated under UV for 30 min, 60 min and 90 min. MBC indicated the lowest concentrations bactericidal effects were observed

UV duration	1-*g*-PMETAC	PPV-*g*-PMETAC (HMw)	PPE-*g*-PMETAC (HMw)	PPV-*g*-PMETAC (LMw)	PPE-*g*-PMETAC (LMw)	Ungrafted PPV	Ungrafted PPE	DMSO
0 min	0	625	833.3	1250	1250	0	0	0
30 min	0	312.5	1250	1250	1250	0	0	0
60 min	0	312.5	1250	1250	1250	0	0	0
90 min	0	156.3	625	2500	625	0	0	0

### 3.3 MIC and MBC of metal ion solutions

To identify metal ion solutions which may improve the antimicrobial ability of the novel compounds, the MIC and MBC of selected metal ion solutions was investigated (Table 4). The most effective metal ion solutions at inhibiting *E. faecium* growth were gold, tin and molybdenum (15.63 μg mL^−1^), whilst palladium, platinum, rhodium, silver and vanadium were also effective (31.25 μg mL^−1^). Metal ion solutions that demonstrated lower inhibitory activity included titanium (62.5 μg mL^−1^), rhenium (78.13 μg mL^−1^), and chromium (125 μg mL^−1^). Nickel, yttrium, copper, bismuth and scandium ion solutions did not display any inhibitory effects (Table 4).

**Table 4:** MIC and MBC (μg mL^−1^) for metal ion solutions against *E. faecium*

Metal ion	MIC (μg mL^−1^)	MBC (μg mL^−1^)
Gold	15.63	62.5
Tin	15.63	31.25
Molybdenum	15.63	31.25
Silver	31.25	250
Palladium	31.25	62.5
Platinum	31.25	62.5
Rhodium	31.25	62.5
Bismuth	46.88	125
Vanadium	46.88	187.5
Gallium	62.5	125
Titanium	62.5	250
Ruthenium	62.5	125
Rhenium	78.13	125
Copper	125	62.5
Yttrium	125	250
Nickel	125	250
Chromium	125	250
Scandium	125	125

In addition to displaying the lowest MIC, tin and molybdenum had the greatest bactericidal properties (31.25 μg mL^−1^), whilst gold, palladium, platinum and rhodium ion solutions (62.5 μg mL^−1^) also demonstrated positive MBC results. Rhenium and copper ion solutions (250 μg mL^−1^) demonstrated weaker bactericidal effects, whilst silver, titanium, yttrium, nickel, chromium, vanadium, gallium, bismuth, and scandium ion solutions showed no activity (Table 4).

### 3.4 FIC synergy of the metals and novel compounds

After identifying the most effective metal ion solutions and compounds, the most inhibitory metal ion solutions, gold tin, molybdenum, silver, palladium, platinum, rhodium, vanadium and titanium were investigated in combination with the four main cationic compounds to determine a possible synergistic relationship between them. None of the combinations caused any antagonistic responses, with all the responses being either additive or synergistic, with the exception of PPE-*g*-PMETAC (LMw) and vanadium ion solution which was indifferent (Table 5). Many of the combinations caused synergistic responses, but only palladium and rhodium ion solutions caused a synergistic reaction with all four compounds. The platinum ion solution was also synergistic and had a positive reaction with three of the compounds excluding PPV-*g*-PMETAC (HMw). Both tin and molybdenum ion solutions, which demonstrated the best MIC and MBC results, had a synergistic reaction with both of the PPV-based compounds and demonstrated additivity with the PPE-based compounds. Finally, the remaining metal ion solutions, gold, silver, titanium and vanadium all demonstrated synergistic reactions with one of the PPV-based compounds and were additive with all the others (Table 5).

**Table 5:** FIC of the activity between the novel compounds and metals tested against *E. faecium*. FIC was calculated using the equation set out in the materials and methods section

	Au	Sn	Mo	Ag	Pd	Pt	Rh	V	Ti
PPV-*g*-PMETAC (HMw)	0.71	0.26	0.26	0.47	0.31	0.60	0.31	0.39	0.41
PPE-*g*-PMETAC (HMw)	0.54	0.54	0.54	0.64	0.43	0.43	0.43	0.74	0.84
PPV-*g*-PMETAC (LMw)	0.38	0.38	0.38	0.64	0.43	0.39	0.39	0.92	0.56
PPE-*g*-PMETAC (LMw)	0.54	0.54	0.54	0.64	0.43	0.43	0.43	1.60	0.84

### 3.5 FBC synergy of the metals and novel compounds

Similarly, to the FIC, the most bactericidal metal ion solutions and compounds were tested at their bactericidal concentrations in combination to determine any synergy in their bactericidal properties. None of the combinations demonstrated any synergistic effects, however, gold, palladium, platinum, rhodium, and vanadium ion solutions all demonstrated additive responses with PPV-*g*-PMETAC (HMw). All other combinations resulted in an indifferent or an antagonistic reaction when in combination with the compounds (Table 6).

**Table 6:** FBC (μg mL^−1^) of the activity between the novel compounds and metal ion solutions

	Au	Sn	Mo	Ag	Pd	Pt	Rh	V	Ti
PPV-*g*-PMETAC (HMw)	1.00	6.00	6.00	1.25	1.00	1.00	1.00	1.00	1.25
PPE-*g*-PMETAC (HMw)	1.17	6.33	6.33	1.33	1.17	1.17	1.17	1.08	1.78
PPV-*g*-PMETAC (LMw)	1.50	7.00	7.00	1.50	1.50	1.50	1.50	0.83	1.50
PPE-*g*-PMETAC (LMw)	1.50	3.00	7.00	2.00	1.50	1.50	1.50	1.25	1.50

## 4. Discussion

All of the conjugated cationic compounds, PPV-*g*-PMETAC (HMw), PPE-*g*-PMETAC (HMw), PPV-*g*-PMETAC (LMw) and PPE-*g*-PMETAC (LMw), demonstrated positive inhibitory and bactericidal properties when tested alone. The effects demonstrated were greater than those of the ungrafted PPV and PPE, indicating that this effect was due to the side chains present on the four cationic compounds. The non-conjugated compound, 1-*g*-PMETAC, demonstrated the least inhibitory effects and no bactericidal effect; for antimicrobial activity to be observed, bottle-brush type conjugated polymers were necessary; this observation is likely due to the high spatial density of cationic ammonium groups as the spatial density of the aforementioned cationic groups was significantly higher in the case of PPV/PPE-*g*-PMETAC (with brush architecture) as opposed to 1-*g*-PMETAC. Sambhy *et al.* (2008) demonstrated that an increase in spatial density by varying spatial positioning and charge/tail ratios of homologous amphiphilic pyridinium polymers significantly increased bacterial membrane-disrupting activities as evidenced *via* antibacterial and hemolytic assays.^24^ This suggested that grafting instead of conjugating the side chains had an impact on its antimicrobial abilities. It has been suggested that the type of binding of the side chains in cationic compounds influences the bioactivity, due to the electrostatic interactions between the compound and the bacterial cell, since cationic surfactants have been found to cause the disintegration of the cell surface of *Escherichia coli*.^25^ Further, under UV conditions, the cationic molecules with the longer side chains demonstrated increased antimicrobial activity than those with the shorter side chains. Similar results have been demonstrated whereby cationic amino acids showed an increased affectivity in longer chain length acids against Gram-positive bacteria.^26^ The effect of polymer conformation, in regards to molecular rigidity, must also be considered. The conjugated polymer backbones utilized throughout this study were expected to enhance the molecular rigidity, and therefore contribute towards the demonstrated antimicrobial efficacy. It has previously been demonstrated that the utilisation of more rigid perfluoroalkyl side chains significantly improved antimicrobial efficacy when compared against a more flexible alkyl side chain.^27^

Similar effects have been noted in the UV activation of riboflavin and its antimicrobial properties against bacteria related to keratitis infections, whereby the results showed there was a significant increase in activity when doubling the UV radiation.^28^ Previous research into the investigation and improvement of antimicrobials has indicated some success with light manipulation.^10,29^ Corbitt *et al.* (2008) developed conjugated polyelectrode capsules produced by layering polycations and polyanions onto a MnCO_3_ template, essentially creating a molecule, which acted as a trap to contain bacteria. When incubated under visible light, this resulted in bacterial cell death, and proved effective against Gram-negative *Pseudomonas aeruginosa*.^4^ In the present study, it is suggested that photo-activation (*via* UV) of the conjugated polymer backbone may have resulted in alternative antimicrobial mechanisms.^30,31^ When utilized alongside the primary antimicrobial mechanism of charged cationic activity, the use of UV may have resulted in an enhanced antimicrobial efficacy. Further, in a study conducted by Wang *et al.* (2013), it was revealed that upon UV light exposure, Gram-negative bacteria (in this case *Escherichia coli*) sustained both cell membrane and cytoplasm damage, whilst Gram-positive bacteria (*Staphylococcus epidermidis*) demonstrated damages to the cell envelope.^31^ It has previously been demonstrated that UV exposure of CPEs can be correlated with the generation of singlet oxygen species (^1^O_2_) and therefore subsequent production of secondary reactive oxygen species (ROS).^3,32^ These results suggested that the way the UV interacts with the compounds may improve the antimicrobial inhibitory ability of certain compound structures. In the cases where a higher MIC value was recorded following a period of UV light incubation, it can be suggested that there was possible degradation of the functional groups present on the cationic polymers which would lead to a reduction in antimicrobial activity. Future studies will be conducted in order to offer a more thorough understanding to this phenomenon. It agreement with our work, it was recently it was demonstrated that manganese-based photoactivatable antibacterial compounds were not necessarily more effective upon UV exposure.^33^

The initial MIC and MBC assays of the metals demonstrated that the most antimicrobial ion solutions against *E. faecium* were gold, silver, palladium, platinum, rhodium, titanium, tin, vanadium and molybdenum. Inhibition by silver was expected as silver ion solution has previously been identified as an effective bacterial inhibitor and has been utilized in many coatings and topical medications, but lacked bactericidal properties when used in ionic form in our study.

When tested for any synergistic inhibitory interactions (FIC), there were a number of positive synergistic reactions between the metal ion solutions and compounds. Any reactions that were not synergistic only additive with the exception of the PPE-*g*-PMETAC (LMw) and the vanadium ion solution which demonstrated an indifferent combination. Overall, PPV-*g*-PMETAC (HMw) was the most synergistic, showing synergy with seven of the nine metal ion solutions tested, closely followed by PPV-*g*-PMETAC (LMw) which demonstrated synergy with six of the nine metal ion solutions. From this, it appeared that the two PPV-based compounds exhibited better synergistic effects than the two PPE-based compounds, PPE-*g*-PMETAC (HMw) and PPE-*g*-PMETAC (LMw), which both only demonstrated synergy with three of the nine metal ion solutions. This might be suggested to be due to the chemical differences in the compound backbone, enhancing the antibacterial efficacy if the compounds. As for the metal ion solutions, the best two were palladium and rhodium, which demonstrated synergy with all the compounds, whilst the metal ion solutions that showed the best inhibitory effects when alone, gold, tin and molybdenum, were only synergistic with a single compound.

When synergy was tested for the metal ion solutions and compounds for their bactericidal effects (FBC), none of the combinations produced any synergistic effects, with most combinations being indifferent. The least two FBC metal ion solutions were tin and molybdenum, which demonstrated antagonistic effects, despite them demonstrating positive results when used alone. Gold, tin and molybdenum ion solutions demonstrated the best inhibitory and bactericidal effects when used alone in this study. Gold has been previously shown to have inhibitory potential in the form of nanoparticles.^34^ The addition of tin complexes to thiosemicarbazones has been shown to improve the compounds overall efficacy;^35^ prior studies with non-cytotoxic molybdenum disulfide nanostructures have also yielded positive results.^36^ Other metals that demonstrated some activity in our study have also been previously shown to demonstrate antimicrobial activity. Vanadium based compounds have previously displayed antimicrobial properties in vanadium peroxidase reactions with *Streptococcus mutans* in planktonic and biofilm form.^20^ Palladium and platinum alloys have also been considered as potential antimicrobial materials for use as medical implants, such as cardiovascular defibrillators or hip and knee implants and catheters.^37,38^ Rhodium complexes with tetraaza macrocyclic and heterocyclic nitrogen ligands have shown effective antimicrobial efficacy against *Escherichia coli* and *Staphylococcus aureus*, particularly against Gram-positive bacteria.^39^ Finally, surface modifications using titanium has been demonstrated to have an antimicrobial effect on *Porphyromonas gingivalis* and *Actinobacillus actinomycetemcomitans*.^40^

## 5. Conclusion

The use of the conjugated bonds in the formation of the cationic molecules increased antimicrobial activity. High Molecular weight (HMW) conjugated compounds had the greatest bactericidal potential on their own and in the presence of UV, which increased the antimicrobial efficacy of some HMW compounds in the short term. The most inhibitory/bactericidal metals were gold, silver, palladium, platinum, rhodium, titanium, tin, vanadium and molybdenum. Following the fractional inhibitory concentrations (FIC)s, palladium and rhodium caused a synergistic reaction with all four compounds whilst all the other metal-compounds demonstrated additive reactions with the exception of PPE-*g*-PMETAC (LMw) and vanadium. Using synergy with the bactericidal concentrations (FBC)s, gold, palladium, platinum, rhodium, and vanadium all demonstrated additive responses with PPV-*g*-PMETAC (HMw). The two PPV-based compounds exhibited better synergistic effects with the metals tested. These results suggest that even when using very similar chemical moieties, the chemical backbone of compounds, alongside the chain lengths and the means of chain attachment all affect the antimicrobial efficacy of a compound. Finally, the importance of the positive results against a potentially AMR organism, *E. faecium*, is significant due to the increase in the incidence of AMR organisms.

## Funding

This research did not receive any specific grant from funding agencies in the public, commercial, or not-for-profit sectors.

## Conflicts of interest

There are no conflicts of interest to declare.

## Supplementary Material
